# News and Miscellany

**Published:** 1903-01

**Authors:** 


### News and Miscellany.

**Married,** at Franklin, Texas, December 21 (ult.), Dr. J. C. Holman to Miss Julia McMillan, all of Franklin.

---

**$800.00 Residence** for sale, on easy terms, and a $2500.00 practice thrown in. Address, T. L., care of TEXAS MEDICAL JOURNAL.

---

**Subscribers** who send me checks on their local banks will please add 10 cents, as it costs me 10 cents on the dollar to cash them here.—F. E. DANIEL, Publisher.

---

**Dr. R. S. Wallis,** of Rockdale, Texas, died suddenly on an I. & G. N. train while en route home, January 7. Dr. Wallis was an eminent physician, and was an ex-confederate surgeon.

---

**The Physician's Protective Accountant** embodies every essential of a perfect and convenient method of accounting for physicians. It may be had of The Clinic Publishing Co., Ravenswood Station, Chicago. Price, complete, $2.00.

---

JONAH, TEXAS, January 1, 1903.

*Dr. F. E. Daniel, Austin, Texas.*

DEAR SIR: —Enclosed you will find post office order for $2.00 for last year's and this year's Red Back. I need it in my business.

Yours fraternally,

B. NOWLIN.

---

**Dr. James Bartlett,** one of the foremost, best, and best known and most popular physicians of San Antonio, died in that city December 13 (ult.), of blood poisoning, the result of picking a pimple on his face with a pin. Dr. Bartlett was formerly of Brenham. He graduated from Medical Department University of Louisville, 1878. A long farewell, delightful, dear friend.

---

**The Following Texas Physicians** have been in attendance at the New Orleans Polyclinic:

Dr. C. G. Salles, Houston; Dr. W. J. Ridgell, Kleburg; Dr. A. B. Edwards, Henrietta; Dr. G. B. Wade, Jacksboro; Dr. J. M. Oxner, Memphis; Dr. A. De Calb Lewis, Cherokee; Dr. F. A. Fuller, Jacksonville; Dr. J. A. Younger, Ballinger; Dr. T. F. Roberts, Brandon.

---

**New Orleans Polyclinic.**—Sixteenth annual session opens November 3, 1902, and closes May 3, 1903. Physicians will find the Polyclinic an excellent means for posting themselves upon modern progress in all branches of medicine and surgery. The specialties are fully taught, including laboratory work. For further information address New Orleans Polyclinic, Post Office Box 797, New Orleans, La.

CHEYENNE, O. T., December 25, 1902.

*F. E. Daniel, M. D., Austin, Texas.*

DEAR DOCTOR:—As a "Christmas Present" I herewith hand you my check for $2.00 as per your statement. Continue the "Red Back" as we could scarcely keep house without it.

After wishing you many more years of happiness and usefulness and a continuation of your great success as an editor, I am,

Very truly and fraternally yours,

J. E. STANDIFER.

---

**"Platform Demand"**—nearly. The State Democratic Convention at Waco in 1901 passed the following resolution:

"Resolved, that we favor the creation of a State Board of Health with provision for the collection of the Vital Statistics of the State, as provided for by the Constitution, Article 16, Section 32, and the enactment of laws to distribute the expense of enforcement equitably between the State, the counties and municipalities."

The people are beginning to see the necessity of preventive measures against diseases other than those of foreign origin. We must have a State Board of Health and Vital Statistics.

---

NEW YORK CITY, December 12, 1902.

*Dr. F. E. Daniel:*

MY DEAR DOCTOR:—A Texas physician wrote me some years ago concerning several cases of fistula in ano which he reported cured by irrigating with hot water. I am anxious to obtain the name of this gentleman, and regret that I have misplaced the letter and forgotten the author's name. Could you help me find him, as he must be well known to you who are personally acquainted with every doctor in Texas, and oblige,

Yours sincerely,

J. A. WYETH.

[Should this meet the eye of the physician referred to he will please write to Dr. Wyeth.—F. E. DANIEL, Editor.]

---

**Washington Post-Graduate Medical School**—Believing that the National Capitol presents many special advantages for post-graduate instruction, the leading members of the profession in Washington City have organized a Post-Graduate Medical School. The course of instruction will consist principally of clinics at the different hospitals of the City and of practical laboratory work. One or two didactic lectures will also be given daily upon such branches as Preventive Medicine, Military Medicine and Surgery, Preventive Inoculations, Serum Therapy, etc.

General Geo. M. Sternberg, U. S. A., has been elected president of the faculty, and the Surgeon Generals of the Army, Navy, Public Health and Marine Hospital Service are members of the Executive Committee.

It is believed that the courses of instruction will be especially valuable for physicians who contemplate entering one of the branches of the public service, also for a general practitioner and for those who intend to practice surgery, gynecology, ophthalmology or laryngology as a specialty. The special attention given to Preventive Medicine and to laboratory work in Bacteriology and Sanitary Chemistry will afford public health officers unusual advantages for perfecting themselves in the scientific studies which serve as a foundation for their particular work.

The first session of the school will commence on Monday, January 12th, 1903. Circulars containing full information will be sent upon application to the President of the Faculty, General Geo. M. Sternberg, U. S. Army, 2144 California Avenue, Washington, D. C.

